# Emerging Issues of Intelligent Assistive Technology Use Among People With Dementia and Their Caregivers: A U.S. Perspective

**DOI:** 10.3389/fpubh.2020.00191

**Published:** 2020-05-21

**Authors:** Deborah Vollmer Dahlke, Marcia G. Ory

**Affiliations:** ^1^DVD Associates LLC, Austin, TX, United States; ^2^TX A&M Center for Population Health and Aging, College Station, TX, United States

**Keywords:** cognitive deficits, intelligent assistive technologies, digital divide, artificial intelligence, agile design, compassionate design, robotics

## Abstract

The increasing number of older adults with cognitive deficits, including dementia, poses a major challenge for public health in the United States. At the same time, the limited number of informal and professional caregivers available to support this rapidly growing population is of mounting concern. Not only does population aging limit the number of potential caregivers, but extant caregivers often lack skills to provide quality care. The integration of intelligent assistive technologies (IAT), including devices, robotics and sensors in many forms, into eldercare, may offer opportunities to reduce caregiver burden and enhance healthcare services while improving the quality of life among older adults with mild to severe cognitive deficits. However, many caregivers and their care recipients lack access to these technologies. The reasons for this reduced access are multifactorial, including the digital divide, sociocultural factors, and technological literacy. This mini review investigates the emerging use of IAT available to caregivers and older adults with cognitive deficits and explores the challenges in socioeconomic status and technological literacy as well as ethical and legal implications that should be considered in the design and development of IAT for older adults with cognitive deficits. Drawing from existing literature, it will suggest frameworks for design and adoption aimed at increased and equitable access for this vulnerable population.

## Introduction

As the global population ages, the population experiencing cognitive deficits including Alzheimer's and related dementias will also expand, resulting in a growing number of older adults with diminished health and well-being that will place undue strains on existing health and supportive services (1, 2). Alzheimer's and related dementias represent a group of symptoms related to cognitive impairments that are sufficiently severe as to interfere with individuals' daily activities. Currently estimated at 5.8 million Americans, the number of persons with Alzheimer's disease is expected to rise exponentially by 2050 when nearly 14 million Americans are projected to have the disease (1). Significant disparities exist in prevalence among population subgroups as defined by race and ethnicity with Black/African American and Hispanics at greater risk compared to older whites (3). Given that increased age is a major risk factor for dementia, it is perceived as predominately a disease of older adults, but early onset is also evident and increasing the overall prevalence and impact (2, 4, 5).

Expanding innovations in technology, including communications, robotics, sensors, and voice activated and intelligent assistive technologies (IAT) are considered promising solutions to address the twin impacts of an aging population with increased cognitive impairment and the shortage of both family and professional caregivers. Ienca et al. developed a systematic review of the spectrum of IATs and their potential for application in dementia care. The list included 539 different devices or systems with the potential for use by those with dementia including those for assistance in the completion of activities of daily living (ADL), systems for cognitive and emotional assistance, health and behavioral monitoring, social interaction and engagement, remote communication, emergency alarm, and mobility aids. Their results suggested that the number of IATs for dementia is exponentially increasing over time, with an average 5-years increase of 400% (6). Table 1 provides a general framework for the variety and types of IAT devices and systems currently available.

**Table 1:** Assistive intelligent technologies devices and systems.

**Category**	**Examples**	**Illustration**
Wearable	Smart watches, necklaces or bracelets with GPS, falls monitoring, and medical monitoring of pulse respiration, oxygen levels etc.	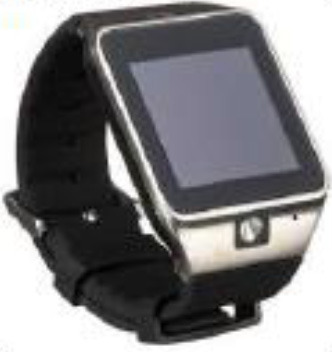
Handheld devices	Tablets, PDAs, GPS trackers, fitness trackers, smart phones etc.	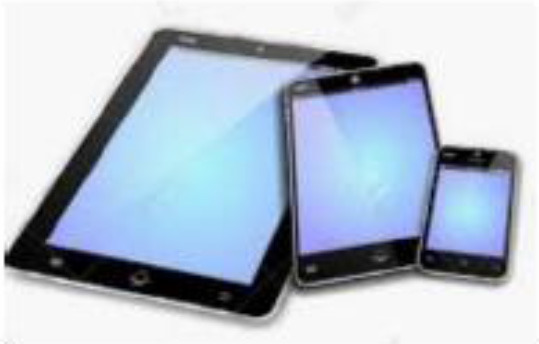
Mobility aids	Smart canes, smart wheelchairs with built in sensors	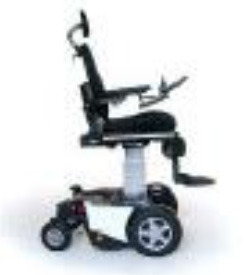
Voice activated assistants	Siri, Alexa/Echo,Google Home	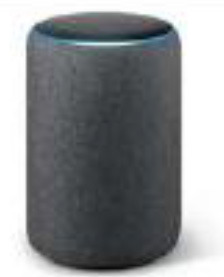
Distributed systems	Smart homes with integrated sensor systems for light, heat, and window coverings	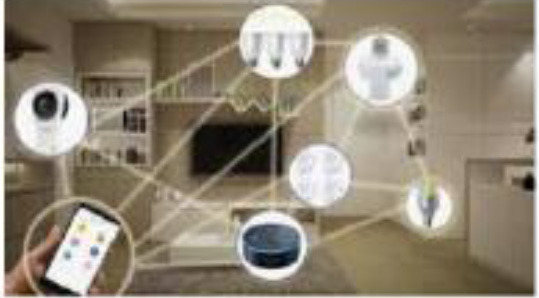
Situation specific robots	Floor cleaning, lifting, soothing, and emotional response	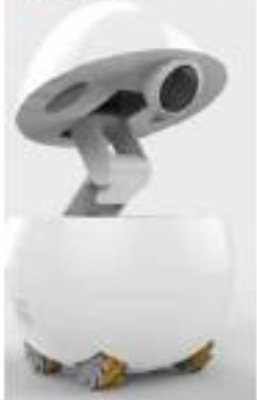

Increasingly, older adults and their family members express their desire to age in place (7). IAT services, robots, and artificial intelligence driven sensors, embedded and wearable devices, including video, and other monitoring systems integrated into the home environment, can offer increased security, safety and health management, and social systems to support both the aging adult with varied levels of cognitive decline as well as family and professional caregivers. While the use of IAT may offer a more economic and efficient means of caring for older adults who wish to age in place, increasingly, caregivers, healthcare providers and ethicists have begun to raise important concerns regarding the design and development of IAT in addition to use and implementation. Fair and equal access to low cost IAT to support adoption across all socioeconomic classes is needed in order to prevent or exacerbate the socioeconomic IAT digital divide. Justice considerations are also at issue in terms of equality, fairness, and openness.

This mini review's objectives are to explore: (1) challenges of access and equity for IAT among caregivers and their care recipients; (2) concerns for inclusiveness in the ethical design and development as well as the use of IAT for and by older adults with varying levels of cognitive decline; and (3) questions of ethics and social values in design and deployment of IAT for dementia care.

It is important to note that this paper is not a systematic review of the literature, nor does it claim to be comprehensive in its coverage. Our goal is to provide a brief review of current research in this area. We lay the framework of standards and ethics to support expanded and equitable access to build awareness and trust in the development and deployment of IAT for use by and among older adults with cognitive deficits. To support these efforts, we conducted a literature search for English language articles indexed in Google Scholar and PubMed databases, We searched title, abstract, and keywords for the terms: “systematic reviews” AND “assistive technology” OR “artificial intelligence” OR “assistive intelligent technology” AND “robotic” AND “Alzheimer's^*^” OR “dementia” OR “aging” OR “elderly^*^.”

## Challenges of Access and Equity for IAT Among Caregivers and Care Recipients

Existing and emerging IAT technologies specific to caregivers and care recipients, especially those with dementia, need to be examined for their potential to assist the entirety of the target population. Due to a number of factors, including socioeconomic status, technological literacy, and the remaining digital divide, a significant number of caregivers and older adults lack access to IAT and the support they might offer. Smartphone ownership, frequently used as an indicator of digital technology adoption, has penetrated to 68% of Americans ages 55–73 and to 40% older adults ages 74–91 (8). However, smartphone ownership appears to be dependent on socioeconomic status. Only 27% of older adults with household incomes under $30,000 own smartphones, compared with 81% of those with more education and higher income status (9). Additionally, there are significant cultural associations in minority race and ethnic status, that combined with lower socioeconomic status and geographic location, suggest a reduced likelihood of IAT adoption among caregivers and care recipients. According to analysis of a Pew Research Center survey of U.S. adults conducted in early 2019, most older adults (74%) ages 55–73 claim to have broadband in their homes, while among those in the 74 to 91 age bracket, only 48% say they have broadband (8).

Despite progress in expanding internet connectivity, ~24 million people in the United States live in “digital deserts” without broadband access, including ~19 million rural Americans and 1.4 million Americans living on Tribal lands (10). Broadband is a key technology platform to deliver many IAT caregiving interventions helpful for persons with dementia such as geofencing sensors and falls monitoring. However, rural populations face significant disparities in access. As compared to non-rural populations, individuals living in rural areas are less likely to receive diagnoses of dementia and more likely to experience preventable hospitalizations (11, 12).

Solutions to the lack of broadband access in rural areas exist. While major internet providers and telecommunications companies balk at the idea of low returns on investment by stringing fiber optic between rural farms and communities, other options are being implemented. The National Rural Electric Cooperatives Association, and many of its rural electric cooperative members, are beginning to build out some rural areas with broadband. However, funding is slow, and many rural areas will remain unserved despite new federal and private sector initiatives^1^. From the private sector, Alphabet Inc., the parent company of Google, is testing internet service from high-altitude helium-filled balloons which may eventually deploy in rural areas of the US. The project is called “Loon” and the Loon balloons can stay aloft for 200 days, providing broadband coverage over an area 20–30 times greater than ground-based systems. Currently, Loon is deploying in disaster response situations in Africa and South America and no timeline has yet been shared for US rural areas^2^.

While broadband distribution may continue to lag for rural residents, increased opportunities for IAT in the form of mobile applications and lower costs for devices to support under-resourced caregivers and care recipients is more encouraging. There are a number of free mobile applications for both iPhones and Android devices that allow older family members and caregivers to stay in touch. Others offer free cognitive stimulation games, puzzles, and photo memory banks. Both Amazon and Google offer their voice-activated virtual assistance devices for under $50 and often bundle them with other services for free. While these devices do require the use of broadband in the home, they can provide older adults with dementia entertainment, access to information and some levels of security (13).

## Challenges of Inclusiveness and Actively Involving People With Cognitive Impairment in the Design of IAT

For older individuals with cognitive impairment, challenges of everyday life become more difficult due to problems with memory, thinking, orientation, language, comprehension, action, and judgement. For nearly all, these impairments only become more problematic over time as the disease progresses for the individual as well as their families, formal, and informal caregivers (14, 15). While IAT in robotics and devices to support aging in place reduce caregiver burden and provide assistance in memory care continue to be developed, there is some concern for low levels of adoption of these technologies. A recent multi-site study with structured qualitative interviews of researchers and healthcare professionals (Wangmo et al.) identified the lack of technological support and digital infrastructure, in addition to with the scarcity of financial and human resources, as major obstacles in successful delivery of older adults and dementia care services (16).

Low adoption rates of assistive technology may signal unmet user needs and wishes in product design. Thus, the principles of agile, open, and user centered design is seen as increasingly important when designing for users with cognitive impairment and dementia (17). The diversity and variety of individuals with cognitive impairment makes it challenging for researchers and innovators to engage in meaningful ways in order to ensure that the IAT being designed is sufficiently flexible to serve that changing needs of the users and their caregivers.

The demands for responsible innovation for IAT extend well-beyond observance and compliance with ethical and value-laden frameworks into ensuring the engagement of a highly diverse group of users. Users may vary in socio-demographics such as race, ethnicity and socioeconomic status as well as the diversity of the condition itself with wide variations in behavioral, cognitive, and emotional aspects. Designing for this group requires engagement with the secondary level of formal and informal caregivers and family members as stakeholders as well as the tertiary stakeholders in clinical and healthcare realms. The benefits of engaging individuals with varying levels of cognitive impairment go beyond ensuring that the designs are “fit for purpose.” The very process of engagement with the designers and innovators with the users and caregivers is likely to have a positive impact because it fosters social interaction, creative expression and builds on the potential for empathic connection among the participants (7). In a systematic review of methods of co-design for technology with people with dementia, Suijkerbuijk et al. and Branco et al. both have identified tools and recommendations from a variety of studies and showed that an increasing number of innovations are being developed with individuals with moderate to severe stages of dementia (18, 19).

Clearly, the engagement in the design process of people with cognitive impairment is not without its challenges. While individuals with mild cognitive impairment usually have no difficulty articulating their thoughts and impressions, as the disease progresses to moderate to severe stages, individuals have more difficulty participating verbally. They also may be experiencing declining health. Further memory issues may make it difficult to establish ethical consent processes for use case and research studies. And, while caregiver input is important, and they represent a critical stakeholder perspective, Wang et al. noted that several of the studies identified the need to separate the individual from the caregiver in the co-design process in order to allow the individuals to express themselves freely (20).

Collaborative design research that includes the input of people with more advanced dementia has most recently included designs for tangible and assistive technologies and communication. The work of Treadaway et al. is especially notable in that their *Compassionate Design* methodology based in LAUGH, an acronym for “Ludic Artifacts Using Gesture and Haptics” is predicated on the understanding that playfulness, fun and laughter benefit well-being of individuals with moderate to severe dementia (19).

The LAUGH research team sought to specifically develop concepts for simple handheld objects to stimulate positive emotions for people living with advanced dementia. According to Zeilig et al. playfulness can be especially important for people with advanced dementia since the disease compromised semantic and declarative memories of events, places and names (20). Ludic or “playful” play is an activity that is in the moment and thus does not rely on the individual's memory of facts or events. And, since it is not goal oriented, there are no judgements of doing things “right or wrong.” While the LAUGH objects were based in technology, they primarily utilized open source technology and low-cost microcontroller systems to imbue the object with elements of sound, light or vibration or to include personalized preferences of the individuals such as music or sounds (19).

## Ethics and Values for Dementia Care: the Need to Be Proactive in Design as Well as Deployment

Dementia care is one of the areas considered most likely to benefit from the current technology transformation in healthcare worldwide for multiple reasons (21–23): (1) the high costs of family and professional care and the promise that IAT might delay or defer the need for institutional services); (2) the diminishing caregiver-to-patient ratio and the potential for IAT and robots to potentially reduce the burden on caregivers and improve quality of care; (3) the, as yet unfulfilled, potential for therapeutic solutions to be derived from insights of artificial intelligence (AI) driven algorithms capturing insights from massive data sets to improve prevention, therapy, and care of aging adults facing cognitive decline; (4) the incorporation of AI and robots in care service to supplement and aid caregivers; and finally, (5) the potential for neuromonitoring and neuro-control devices to provide personalized and patient-centered care solutions to delay further impairment or to provide memory and engagement support. The Wangmo et al. research also explored the ethical challenges of IAT designed to be used by vulnerable individuals. Those interviewed suggested that age and multi-morbidity-related frailty and cognitive disability are vulnerable especially when the technologies involved machine intelligence, collected sensitive data or were operated in close proximity to the human body (16).

According to the International Federation for Robotics (IFR), more than 4.7 million service robots were active for personal and domestic use in 2014, including a 542 percent increase in assistive robots for the elderly and disabled with rapidly growing market penetration (24). Other estimates suggest that by 2018, ~35 million service robots –defined as those which “performs useful tasks for humans or equipment excluding industrial automation application.” will be active (24).

A recent systematic review by Sriram et al. explored the use of IAT by caregivers of those with dementia and the impact of these technologies on carer's lives with the recommendation for a standard and person-centered system be developed (25). A scoping review by Øksnebjerg et al. focused on the need for high-quality research into essential aspects of delivering applicable, effective, and sustainable assistive technology to support self-management of people with dementia suggesting the need for evidence-based methods to promote and qualify user involvement, dissemination, and adoption (26). For the most part, this current research articulates concerns for ethics issues such as privacy, security and consent have occurred ex post of the implementation or in the process of use. While concerns about the implications for technology in dementia care surfaced nearly 20 years ago (27–30), only recently have literature reviews in this area begun to highlight the need for ethical frameworks and value-centered innovation in the initial design of IAT for dementia care.

In part, this increased awareness for user-centered design may be attributed to the aging population's existing familiarity with the Internet and the growing global use of AI and digital technologies, which is speeding adoption and also driving innovation. The concerns also stem from the consideration that the ubiquitous and somewhat pervasive nature of IAT affect not just the physical and clinical aspects of the users, but also the emotional, psychosocial, behavioral, and relational aspects as well. The concept that companionship robots or even digital applications available on a tablet might alleviate isolation, agitation or improve the emotional well-being of elders with cognitive deficits has both positive and concerning implications.

Increasingly researchers are questioning what the unintended consequences may be of switching from solely human care to a mix of human and technology assisted care (27). In addition to the ethical and value-based implications of IAT, there are concerns about the extent to which laws and regulations may be needed in order to protect the human rights of this vulnerable group of users. These considerations are well-articulated in a framework developed under the United Nations Convention on the Rights of Persons With Disabilities which posited that it is “critically important for developing a robust, rights-focused regulatory framework for the use of assistive technologies in dementia care (31).” In Winfield and Jirotka's (32) paper on ethical governance and the need for building trust, they argue that with the increasing pace of innovation, new and more agile governance processes are needed so that not only policy makers, but also businesses and innovators feel the duty to engage with the ethical and value-based responsible innovation of IAT, with a focus on Artificial Intelligence (AI) and the near future development of autonomous AI systems (32).

Among the many guidelines and frameworks for IAT, the British Standard's BS8611, is perhaps one of the broadest and far reaching as it articulates a range of ethical hazards and potential mitigation for societal, commercial, financial, and environmental risk (33). It also provides innovators and designers of IAT and robotic systems guidance on how to assess and reduce such societal risks as loss of trust, deception, privacy, security, safety, and confidentiality as well as such future concerns of addiction and unemployment. While acknowledging that there is no shortage of sound ethical principles being espoused for IAT, Winfield and Joitka suggest that there is little evidence that these principles are being translated into practice.

**Figure 1:**
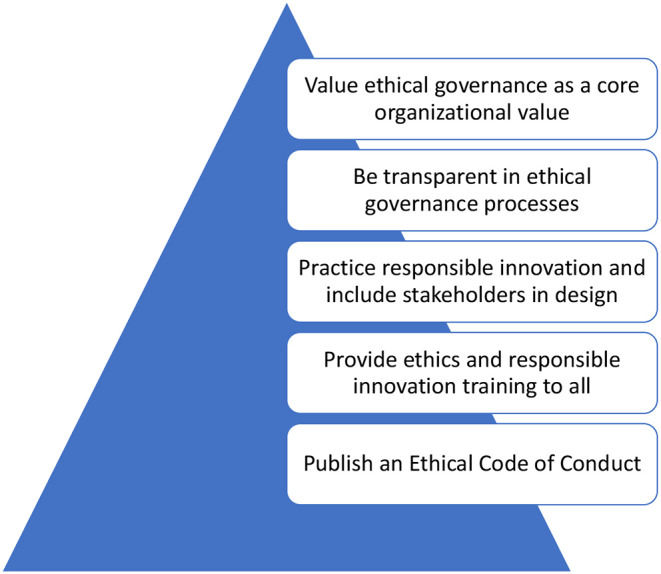
Five pillars of ethical governance (29).

To further the practice of strong ethical governance in organizations and institutions, they suggest five pillars as summarized in Figure 1: (1) Publish an *ethical code of conduct* so that everyone in the organization understands what is expected of them; (2) Provide ethics and *Responsible Innovation* training for everyone, without exception; (3) Practice *Responsible Innovation*, including the wider engagement of stakeholders based in the BS8611 and new process standards such as IEEE P700 Model Processes for addressing ethical concerns during system design (still in draft); (4) Be transparent in ethical governance, meaning transparency of process, not product; and lastly (5) Value ethical governance in the organization such that it is a core value, not just a smokescreen for values like maximizing shareholder returns (32).

## Conclusion

IATs' are increasingly supporting the desire for aging in place and independence of people living with a range of cognitive impairments allowing them and their caregivers to live healthier and potentially more engaged lives. However, to meet the goals for IAT to be developed more broadly in a highly accessible, ethical, compassionate, and just manner, it is essential that the technologies be affordable and usable. While there are an increasing number of low-cost devices and free mobile applications that can be useful to both caregivers and care recipients with dementia, most of these require a broadband connection or at the very least, a smartphone. This requirement continues to leave out a broad swath of those who live in rural areas and whose socio-economic status doesn't support broadband or regular cellular service as well as those whose cultural, racial or ethnic situations are not supportive of the technological literacy needed to use these devices and tools.

Our mini review of the current research suggests that increasingly the developers of IAT tools and technologies are becoming more cognizant of the need to engage with both the caregivers and the potential older users, including those with early stages of dementia in the design and development of tools and systems that may be helpful and useful for them. While we note that the majority of the citations are US-based, and hence limit our generalizability, we believe that many of these issue are of concern worldwide, as indicated by the attention being paid by international and national organizations such as the World Health Organization (31) and scientific organizations in other countries (32, 33) We also found support for this in several recent articles targeted at technology designers and developers (21–23).

It is critical that attention be paid when a technology or device is no longer desirable for a person whose dementia may have progressed. Our mini review is concordant with several current systematic reviews addressing the need for frameworks, guidelines, and regulations for IAT adoption among caregivers and those with dementia (21–23). In addition to the need for increasing access among rural and lower socioeconomic populations, it is imperative that these ethical and value laden systems for responsible innovation be implemented collaboratively and compassionately. The potential for IAT to be beneficent in supporting independence, security, and safety of people with dementia is clear. However, IAT can also be implemented in a way that is detrimental to the rights, privacy, dignity and freedom of people living with dementia. Thus, attention and diligence must continue to be observed in the ongoing development and evaluation of IAT so that the frameworks which support human rights and equity are always foremost and truly support the optimal quality of life for those living with cognitive deficits. Finally, concerns related to equitable access to and use of IATs' requires further research in understanding personal, ethical, ideological, social and cultural concerns, and possible mechanisms of addressing them. A recent article by Lindeman et al. provides guidance for such research (34).

## Author Contributions

DV and MO contributed to the conceptualization and preparation of this manuscript. All authors edited and approved the final draft.

## Conflict of Interest

DV was employed by the company DVD Associates, LLC. The remaining author declares that the research was conducted in the absence of any commercial or financial relationships that could be construed as a potential conflict of interest.

## Footnotes

^1^Available online at: www.mcclatchydc.com/news/nation-world/national/economy/article226599354.html (accessed March 19, 2020).

^2^Available online at: medium.com/loon-for-all (accessed March 20, 2020).
